# Lowest Environmentally Relevant Concentrations of Ionic Silver in Picograms per Liter Impair Life History Traits and Population Growth of *Daphnia magna* (Cladocera)

**DOI:** 10.3390/jox16020060

**Published:** 2026-04-02

**Authors:** Jingyun Ding, Stefanie Krais, Zequn Li, Rita Triebskorn, Heinz-R. Köhler

**Affiliations:** Animal Physiological Ecology, University of Tübingen, Auf der Morgenstelle 5, D-72076 Tübingen, Germany; stefanie.krais@uni-tuebingen.de (S.K.); zequn.li@student.uni-tuebingen.de (Z.L.); rita.triebskorn@uni-tuebingen.de (R.T.); heinz-r.koehler@uni-tuebingen.de (H.-R.K.)

**Keywords:** *Daphnia magna*, silver, Ag^+^, population growth, multigenerational toxicity, transgenerational effects, aquatic toxicology

## Abstract

Although chronic contamination by silver ions (Ag^+^) can persist in aquatic systems over long periods of time and can therefore have an impact on population developments, regulatory testing commonly relies on single-generation endpoints. Here, we used *Daphnia magna* to quantify long-term effects of pg/L to ng/L concentrations of Ag^+^ across generations and to test whether recovery depends on exposure history. Using 21 d life-cycle assays over up to seven consecutive generations, we quantified survival, key life-history traits, and population fitness (intrinsic rate of natural increase, *r*). In our study, low environmental concentrations of Ag^+^ caused minimal mortality, but sublethal effects persisted or multiplied over generations. Notably, continuous exposure led to significant reductions in body length and *r* at 50 pg/L (nominal LOEC) by the fourth generation exposed, representing population-relevant effects of Ag^+^ at very low concentrations which should be given consideration in the assessment of both water quality and the chemical itself. Recovery was concentration-dependent: low-concentration-exposed lineages recovered within a few generations, whereas 15 ng/L exposure resulted in persistent deficits even through the recovery period of three generations. Exposure-history patterns indicated that long-term outcomes were dominated by the cumulative number of exposed generations. These findings highlight the limitations of acute and single-generation assays and emphasize the importance of considering information on the effects of chemicals, including Ag^+^, across multiple generations in risk assessments. They also highlight the need to include expectations regarding recovery after the removal of pollutants in these assessments.

## 1. Introduction

Silver (Ag) finds extensive applications in diverse sectors, such as electronics, battery manufacturing, catalysis, and jewelry production. The environmental release of Ag, particularly in its ionic form (Ag^+^), is a significant ecological concern due to its high toxicity to aquatic life, which can be triggered at trace concentrations ranging from ng/L to μg/L [1,2,3,4,5,6]. Total Ag concentrations in natural waters fluctuate between 0.03 and 500 ng/L [7], and levels of dissolved Ag^+^ concentrations generally remain below 0.2 μg/L [8]. In addition to traditional applications, Ag^+^ has been characterized as a potent and highly efficient biocide against a broad spectrum of microorganisms coupled with relatively low toxicity to mammals [9]. The combination of high antimicrobial activity and a favorable mammalian safety profile make Ag^+^ a viable alternative to conventional biocides in material applications, such as coatings and polymers [9,10]. Industry practice reflects this preference: a survey of biocidal product manufacturers indicated that approximately 91% of silver used in such formulations is in Ag^+^ [11]. A significant portion (e.g., 15%) of anthropogenic silver entering European waters originates from such biocidal products like textiles and plastics [12], thereby linking consumer usage directly to environmental exposure.

Particularly in recent years, the applications of Ag as a biocide further increased during the COVID-19 pandemic due to Ag-based disinfectants, masks, and functional textiles [13]. Additionally, increased littering of personal protective equipment (PPE) also emerged during the COVID-19 pandemic [14]. As of 2025–2026, silver-based biocidal products and treated materials remain environmentally relevant, as indicated by ongoing regulatory attention and continued research [15,16,17]. These recent developments have undoubtedly added to baseline sources, contributing to additional Ag^+^ contamination of aquatic environments and threatening the ecological balance of these ecosystems. The ionoregulatory toxicity of Ag^+^ in aquatic species, involving the inhibition of the Na^+^/K^+^-ATPase pump, underscores its high hazardous potential [18]. Thus, the regulatory status of Ag reflects its significant environmental concern. It is also considered a priority substance regulated under the European Commission Biocidal Products Directive and is designated a priority pollutant by the US Environmental Protection Agency (U.S. EPA) [12,19].

Recently, Mertens et al. [20] provided further justification for read-across from soluble Ag salts to silver nanoparticles (AgNPs). They confirmed that Ag^+^ can be used as a conservative estimate for the aquatic effects of AgNPs at comparable Ag concentrations. Many current regulatory frameworks for regulating Ag pollution are also primarily based on research data concerning the ionic form, Ag^+^ [21,22]. However, the ecotoxicological evidence base for Ag is largely concentrated on silver nanomaterials, particularly AgNPs. Multiple cross-taxa reviews have systematically synthesized AgNPs effects and mechanisms across bacteria, plants, aquatic invertebrates (including zooplankton), and fish, indicating that AgNPs-focused studies remain predominant within the ecotoxicology research [23,24,25], and the available evidence is still largely derived from OECD standard acute or short-term single-generation assays [26,27,28,29]. By contrast, long-term multigenerational and real transgenerational assessments of Ag^+^ are still rare. To our knowledge, the few existing long-term studies of Ag^+^ on toxicological models involving invertebrates [30,31,32,33,34] have predominantly focused on nematodes [31,32,33]. In light of the increased use and environmental release of Ag^+^ during the pandemic, and given the evidence that AgNO_3_-based Ag^+^ testing provided a conservative estimate of the aquatic effects of AgNPs at comparable Ag concentrations [20], it is clear that further research is needed. This indicates that a systematic and comprehensive long-term, multigenerational toxicity risk assessment of Ag^+^ in aquatic organisms is essential [34]. This would provide more reliable data for establishing regulatory standards for Ag in fresh waters.

*Daphnia magna* is a key component of freshwater food webs, feeding on phytoplankton and serving as a major prey species for invertebrate and vertebrate predators [35]. In addition, *D. magna* is one of the well-established model organisms in aquatic ecotoxicology. It is used in international OECD standardized tests for acute immobilization [26] and 21-day reproduction [27], and its life-history traits provide sensitive indicators of contaminant stress [26,27,36]. Multigeneration studies have also shown that *D. magna* is well suited for evaluating population-level responses to metals and other stressors [37,38,39]. In addition, its parthenogenetic reproductive mode, producing genetically identical offspring, provides a unique advantage for multigenerational experimental designs by minimizing genetic variance and allowing clear attribution of observed effects to the treatment rather than genetic drift [40]. In this context, it is essential to differentiate between multigenerational effects (observed during continuous exposure) and transgenerational effects (persisting in descendants never directly exposed) to fully understand a contaminant’s impact [41,42]. A central question in long-term toxicity research is whether altered sensitivity results from reversible physiological acclimation; from stable, heritable epigenetic modifications; or from the selection of tolerant genotypes. Conducting recovery experiments, where exposed lineages are transferred to clean environments for a long period, is a key strategy to discriminate between these potential mechanisms [31,40]. Research has exemplarily addressed multigenerational metal toxicity in *D. magna* [40,43] and suggested metals to induce epigenetic changes like DNA methylation [44].

However, when it comes to studies of Ag on *D. magna* specifically, the available investigations still largely rely on traditional acute assays for Ag^+^ and AgNPs [45,46,47,48], but few chronic tests for AgNPs [30,47,49,50]. By comparison, the more limited number of chronic Ag^+^ studies in *D. magna* have typically used a range of comparatively high concentrations and/or single-generation tests, thus limiting their environmental relevance [21,46,47,51]. Moreover, the current research has not comprehensively addressed how different exposure histories (e.g., continuous vs. intermittent, and early vs. late exposure) modulate toxicity outcomes and recovery potential, a critical consideration given the episodic nature of many pollution events [52]. Furthermore, direct comparisons designed to disentangle the effects of exposure duration on the persistence of toxicity into recovery generations are rare but essential for identifying exposure thresholds that trigger irreversible population-level consequences. In summary, a systematic investigation into the multigenerational and real transgenerational toxicity of Ag^+^ at low, environmentally relevant concentrations in the ng/L or even pg/L range in *D. magna* is still lacking.

To address these gaps, we designed a comprehensive long-term study to investigate multigenerational and real transgenerational responses (in recovery) of *D. magna* to sublethal Ag^+^ concentrations in the ng/L and pg/L range using three complementary experimental series. First, we established a series of solutions with nominally increasing concentrations (0, 0.05, 0.5, 5, and 15 ng/L Ag^+^). These solutions were used to cultivate four generations of exposed populations (F0–F3) and three subsequent generations of recovery in clean medium (F1’–F3’). This approach enables the quantification of multigenerational effects of exposure and real transgenerational effects during recovery at environmentally relevant Ag^+^ levels. Second, we examined how different four-generation exposure histories at a relatively high environmentally relevant concentration (15 ng/L Ag^+^) influence life-history traits and population growth, including continuous, intermittent, early, and late exposure patterns. Third, we directly compared a one-generation exposure followed by three recovery generations, with a four-generation exposure followed by the same number of recovery generations to distinguish the effects of short versus prolonged exposure history on transgenerational responses. Across all series, we measured survival; reproduction (time to first reproduction, number of broods, average offspring number per brood, and total offspring number per surviving adult); growth (body length); and population fitness, i.e., the intrinsic rate of natural increase (*r*), to provide an integrated risk assessment of how low concentrations of Ag^+^ affect *D. magna* populations over time and how reversible these effects are once exposure stops.

## 2. Materials and Methods

### 2.1. The Model Organism and Culture Conditions

The *Daphnia magna* STRAUS, 1820 (Crustacea, Cladocera), used in this study originated from a healthy laboratory stock maintained in the Laboratory of the Animal Physiological Ecology group, University of Tübingen (Germany). The stock population was cultured in a climate chamber using ISO test medium [53] at 20 ± 1 °C under a 16 h/8 h light/dark photoperiod. Half of the culture medium was renewed twice per week. Daphnids were fed a suspension of the green alga *Scenedesmus subspicatus*.

### 2.2. Test Chemical

Silver nitrate (AgNO_3_; purity ≥ 99.0%) was obtained from Sigma-Aldrich (Merck, Darmstadt, Germany). A stock solution was prepared by dissolving 500 mg of AgNO_3_ in 1 L of deionized water. Test solutions were prepared as ISO medium [53]. Briefly, four salt stock solutions were prepared in deionized water: CaCl_2_·2H_2_O (11.76 g/L), MgSO_4_·7H_2_O (4.93 g/L), NaHCO_3_ (2.59 g/L), and KCl (0.23 g/L). For each liter of ISO medium, 25 mL of each stock was added to 900 mL deionized water and brought to 1 L with deionized water. The prepared ISO medium was aerated prior to use and equilibrated to the experimental temperature (21 ± 0.5 °C) prior to use. In order to minimize potential stress or disturbance to the organisms during the long-term experiments, we aimed to balance environmental stability with limited handling [34]. All exposures were conducted in a dedicated, clean indoor climate chamber reserved exclusively for this experiment, minimizing potential airborne or cross-experimental contamination. All Ag^+^ test solutions are reported as nominal concentrations, and the final test solutions derived from an AgNO_3_ stock solution were diluted with ISO medium. This approach was adopted because the multigenerational design required frequent medium renewals (every other day) and involved numerous exposure vessels, which made chemical analysis of the medium essentially impossible for each exposure and each generation after each medium change. In addition, routine quantification of Ag at the pg/L–ng/L range across frequent renewals and multiple treatment groups would require ultra-trace analytical work-flows with stringent contamination control and preconcentration/enrichment steps, particularly in ionic matrices where interferences and organic ligands can affect Ag determination. Such workflows are feasible for targeted environmental monitoring but were not practical at the scale of the present multigenerational design [54,55]. To minimize deviation of real test concentrations from nominal values, both the silver nitrate stock solution and test solutions were protected from light exposure and stored in brown bottles wrapped in aluminum foil. Prior to each medium renewal, the test Ag^+^ medium was freshly prepared using aerated standard ISO culture medium under conditions identical to experimental conditions, and it was allowed to equilibrate for 24 h. Similar long-term studies on silver also have relied on nominal concentrations without routine chemical analysis, explicitly acknowledging Ag^+^ stability [34]. Other multigeneration studies on *D. magna* did apply diluted AgNO_3_ test solutions and reported nominal concentrations as well [30]. Given the ionic composition of ISO medium, complexation and precipitation processes may be expected to reduce the freely available Ag^+^ fraction relative to nominal values; thus, nominal concentrations in the present study should be interpreted as a conservative worst-case representation of exposure.

### 2.3. Experimental Design

**Figure 1:**
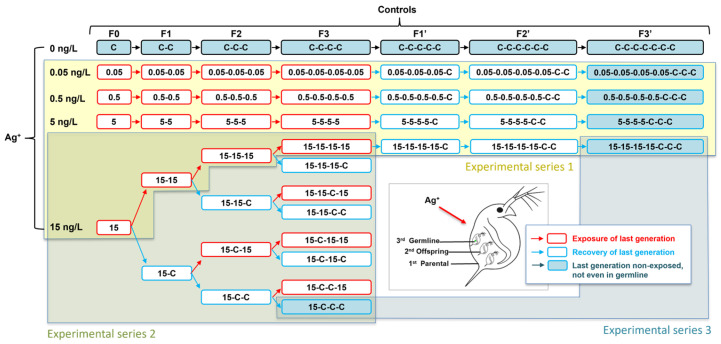
Overall long-term experimental design for multigenerational and transgenerational exposure of *Daphnia magna* to silver ions (Ag^+^).

Study objectives and overall conceptual framework: this study aimed to examine long-term multigenerational effects of environmentally relevant silver ions (Ag^+^) in *Daphnia magna*, with a focus on (1) potential cumulative toxicity under long-term low-concentration exposure, and (2) how different exposure histories (timing and patterns) influence the animals’ recovery potential. To achieve this, we implemented an integrated experimental design consisting of three parallel experimental series spanning seven consecutive generations (F0–F3 (Ag^+^-exposure phase) and F1’–F3’ (recovery phase)). Specifically, the design included the following: (1) a stable, environmentally relevant concentration gradient of Ag^+^ in the pg/L and low ng/L range with a subsequent recovery phase; (2) effects linked to different exposure histories at a specific, relatively high environmentally relevant Ag^+^ concentration (15 ng/L); and (3) a targeted comparison of short versus prolonged exposure history, starting with an exposure of 1–4 generations to 15 ng/L Ag^+^. Approach (3) was actually a comparison of selected exposure series conducted in experiments (1) and (2) (Figure 1). All experiments were initiated simultaneously.

Each horizontal track represents a specific exposure history, referring to the clonal populations that were continuously propagated from the F0 to the F3 generation (and extended to the F1’–F3’ generations where applicable) under each Ag^+^ exposure and clean medium conditions. Rows refer to the tested Ag^+^ concentrations (0, 0.05, 0.5, 5, and 15 ng/L as AgNO_3_); columns refer to the different generations. Each box shows the exposure history of a lineage as an up-to-seven-character code, where numbers denote exposure to the corresponding Ag^+^ concentration (ng/L), and ‘C’ (control) denotes culture in clean ISO medium. Experimental series 1 (yellow background) comprises the concentration-gradient lineage that experienced four consecutive generations of exposure (F0–F3, followed by three generations in clean medium to assess recovery and transgenerational effects (F1’–F3’). Experimental series 2 (greenish background) comprises 15 ng/L lineages with different four-generation exposure histories (continuous, early, late, and intermittent exposure). Experimental series 3 (bluish background) compares a single-generation exposure followed by three recovery generations (15-C-C-C), with a four-generation exposure followed by the same recovery period (15-15-15-15-C-C-C). The schematic depiction of *D. magna* in the bottom-right corner illustrates the transgenerational levels of parental generation, offspring generation, and germline. The labels classify the respective exposure situation as ‘last generation exposed’; ‘last generation recovered’; or ‘last generation non-exposed, not even in the germline’.

#### 2.3.1. Experimental Series 1: Environmentally Relevant Ag^+^ Concentration Gradient with Multigenerational Exposure and Recovery (F0–F3; F1’–F3’)

This series represents long-term exposure under an environmental concentration gradient, followed by transfer to clean medium to evaluate recovery across generations. Prior to the long-term experiment, a 48 h range-finding test was conducted following the OECD *Daphnia* sp. Acute Immobilisation Test Guideline [26] to identify a suitable sublethal concentration range for chronic multigenerational testing. Neonates (<24 h old) were collected from the stock culture to standardize age at test initiation. Nominal Ag^+^ concentrations for the acute OECD 202 test [26] were 0, 0.1, 0.5, 1, 2, 3, 5, 7, and 10 µg/L (as AgNO_3_). Immobilization was recorded at 24 h and 48 h, and the resulting EC_10_ (48 h) value of Ag^+^ (14.71 ng/L, nominal) was used as a reference point for setting the highest long-term test concentration. This EC_10_ level for acute toxicity is close to the EU annual-average EQS level (AA-QS_eco,fw_ = 0.01 µg/L) [21,56].

Based on the range-finding results, five nominal Ag^+^ concentrations were used for the 21 d life-cycle tests: 0 (control), 0.05, 0.5, 5, and 15 ng/L. Each concentration group was exposed for four consecutive generations (F0–F3). After completion of F3, lineages were transferred to clean ISO medium (without AgNO_3_) and maintained for three additional generations (F1’–F3’) to quantify recovery. Lineage naming and generation codes are shown in the experimental conceptual diagram (Figure 1).

The operation procedure of our long-term study was comparable to the method used by Völker et al. [30] and Jacobasch et al. [57]. We exposed the parental generation of *D. magna* over a period of 21 days and always used the third brood to start the next respective generation. For every generation, 10 replicates per treatment were exposed. In each replicate, a single offspring (<24 h) was placed in a glass beaker (50 mL), filled with 40 mL of test medium. Test media were changed every two days. The *Daphnia* specimens were fed every other day with suspensions of the green algae *S. subspicatus* (8 × 10^5^ cells mL^−1^). The number of offspring was counted and removed every day. Long-term experiments were conducted under identical conditions for both experimental and control groups: temperature was maintained at 20 ± 1 °C, with a light–dark cycle of 16 h/8 h (light/dark). Experimental series 2 and 3 had different experimental designs (see below) but otherwise employed the same operational procedures and conditions as experiment series 1. Three series of experiments were initiated simultaneously.

#### 2.3.2. Experimental Series 2: Exposure-History Patterns at 15 ng/L Ag^+^ Across Four Generations (F0–F3)

This series simulates field-relevant situations where contamination may be inconstant, including intermittent discharges and different timings of transfer to clean medium. The aim was to quantify how life-history endpoints vary with exposure history and recovery timing, and to test whether response patterns are better explained by the total number of exposed generations or by the specificity (early vs. late) of the generations that were exposed.

A single nominal Ag^+^ concentration (15 ng/L; as AgNO_3_) was used to construct multiple four-generation exposure patterns (F0–F3). Each pattern is designated by a four-character code (e.g., 15-15-C-15), where C indicates clean ISO medium. To generate the exposure-history design, offspring from the F0 15 ng/L group were used to start F1 and were randomly assigned to either re-exposure at 15 ng/L or to transfer to clean medium, producing two F1 lineages (15-15 and 15-C). The same branching procedure was applied in each subsequent generation, resulting in a total of 15 exposure-history groups (1 × F0 + 2 × F1 + 4 × F2 + 8 × F3) plus four control groups. Each replicate consisted of one individual maintained in a 50 mL beaker with 40 mL Ag^+^ test medium accordingly, with 10 replicates per exposure-history group. For each lineage, the next generation was initiated using neonates from the third brood, pooled within the same treatment lineage and then randomly allocated according to the planned exposure-history pattern (Figure 1).

#### 2.3.3. Experimental Series 3: Short Versus Prolonged Exposure History with the Same Recovery Period (15-C-C-C vs. 15-15-15-15-C-C-C)

This approach is based on exposure series of experiments 1 and 2 and contrasts two realistic scenarios: (1) a short contamination event followed by sustained clean condition, and (2) prolonged contamination followed by transfer to clean medium. The purpose was to distinguish between the strengths of persistence of possible trans-generational effects caused by single-population exposure vs. those deriving from multigenerational exposure. Two exposure histories were compared: 15-15-15-15-C-C-C (four consecutive exposed generations followed by three clean generations) and 15-C-C-C (one exposed generation followed by three clean generations). Both histories were assessed after the same number of clean generations, so differences among histories cannot be attributed to unequal recovery time (Figure 1).

### 2.4. Life-History Endpoints in Chronic Toxicity Tests with Ag^+^

Life-history endpoints were measured across generations, with a focus on survival, reproduction, growth, and population-level fitness, following the general structure of the OECD guideline for 21 d reproduction tests [27]. Adult survival of parental females was recorded daily for each generation. Reproductive endpoints included the time to first reproduction per surviving female, the number of broods per surviving female, the total number of offspring per surviving female within 21 days, and the average number of offspring per brood (i.e., offspring per brood averaged per surviving female). The body length of surviving adults was measured at the end of each 21 d test. Body length was measured from the apical end of the compound eye to the tail–spine base (excluding the length of the spine) [58]. Population fitness (intrinsic rate of natural increase, *r*) was calculated from life-table information using the Euler–Lotka equation and solved numerically for each treatment lineage, as commonly applied in multigenerational *Daphnia* studies [38].

$$1=\sum_{x=1}^{n}l_{x}\;m_{x}e^{-rx},\;$$

where *l_x_* is the fraction of individuals surviving until age *x*, *m_x_* is the number of offspring produced per surviving female between ages *x* and *x* + 1, and *r* (day^−1^) is the intrinsic rate of natural increase.

### 2.5. Data Analysis

All statistical analyses were performed using SPSS 22.0. Endpoints were analyzed for normality (Shapiro–Wilk test) and homogeneity of variance (Levene’s test). When assumptions were met, differences among groups were tested using Student’s *t*-test (two-group comparisons) and one-way analysis of variance (ANOVA) followed by Fisher’s least-significant-difference (LSD) multiple comparisons. When assumptions were not met, non-parametric tests were applied, including the Mann–Whitney U test (two-group comparisons) and the Kruskal–Wallis test (multi-groups), followed by Bonferroni correction for pairwise comparisons. The significance level was set at *p* < 0.05.

## 3. Results

### 3.1. Survival

In all three experimental series, survival remained high throughout the 21 d assays across generations and treatments (Supplementary Tables S1 and S2). Survival was therefore not considered a primary driver of treatment-related variation in reproduction, growth, or population growth rate under the tested pg/L and ng/L Ag^+^ exposure conditions.

### 3.2. Results for Experimental Series 1: Effects of Environmentally Relevant Ag^+^ Concentration Gradient with Multigenerational Exposure and Recovery (F0–F3; F1’–F3’)

This experimental series represents long-term Ag^+^ contamination at environmentally relevant concentrations, followed by transfer to clean medium. The aim was to quantify cumulative multigenerational toxicity across a concentration gradient (0–15 ng/L) and to characterize recovery trajectories after Ag^+^ removal. A four-generation exposure to Ag^+^ (0, 0.05, 0.5, 5, and 15 ng/L) was conducted (F0–F3), followed by a three-generation recovery period in clean medium (F1’–F3’).

#### 3.2.1. Time to First Reproduction (Figure 2A)

Ag^+^ delayed the time to first reproduction in a concentration- and generation-dependent manner, and the effect sizes among treatments increased after successive exposure generations. Delays were small in F0 but became clearer with accumulated exposure. At 15 ng/L, significant delays occurred from F1 onward, with the largest delay in F3. Non-significant delays also emerged at lower concentrations later in the exposure series (notably 0.5 and 5 ng/L in later exposure generations), but these effects were less persistent. During recovery (F1’–F3’), the 15 ng/L lineage remained significantly delayed relative to controls, while the tendencies to delays at 0.5 and 5 ng/L resolved within one or two recovery generations.

**Figure 2:**
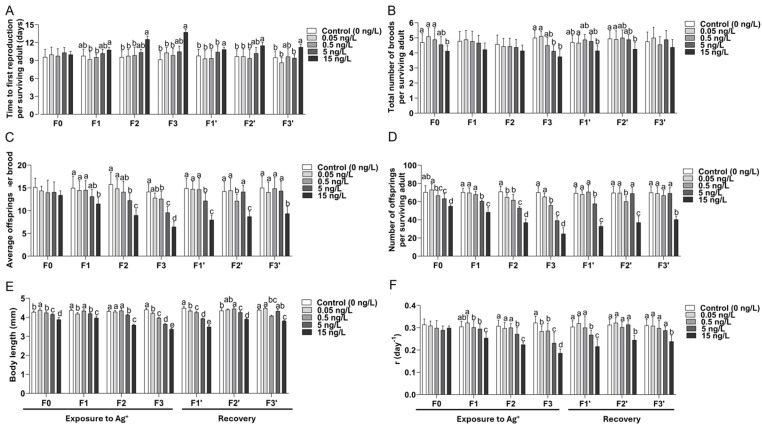
Multigenerational and recovery responses of *Daphnia magna* to a concentration gradient of Ag^+^ (experimental series 1). Means (+SD) for life-history traits across four exposed generations (F0–F3) and three recovery generations in clean medium (F1’–F3’) at 0, 0.05, 0.5, 5, and 15 ng/L Ag^+^. (**A**) Time to first reproduction per surviving adult (days). (**B**) Total number of broods per surviving adult. (**C**) Average offspring number per brood. (**D**) Total number of offspring per surviving adult over 21 d. (**E**) Body length (mm) at the end of the test. (**F**) Intrinsic rate of natural increase (*r*, day^−1^). For (**A**–**E**), n corresponds to the number of surviving adults in each treatment and generation (see Supplementary Table S1). For (**F**), *r* was calculated by including all individuals (surviving and dead), giving n = 20 for controls and n = 10 for each Ag^+^ treatment. Within a given generation, differences among concentrations in (**A**,**B**) and body length in F3’ in (**E**) were analyzed by Kruskal–Wallis test, followed, when significant, by Bonferroni-corrected pairwise comparisons. Differences among concentrations in the remaining traits and generations ((**C**–**F**), except F3’ in (**E**)) were analyzed by one-way analysis of variance (ANOVA), followed, when significant, by Fisher’s least significant difference (LSD) multiple comparisons. Different letters above bars indicate significant differences among the different groups, characterized by the respective exposure concentrations, within a given generation (*p* < 0.05).

#### 3.2.2. Total Number of Broods per Adult (Figure 2B)

Brood number was comparatively stable at lower concentrations but declined under higher concentrations, particularly after multiple exposure generations.

In F0, the 15 ng/L group produced significantly fewer broods than controls and lower-concentration groups. Differences were smaller or absent in F1–F2, but by F3, the two highest concentration groups (5 and 15 ng/L) tended to produce fewer broods than controls. During recovery, the 5 ng/L lineage returned to control levels within one recovery generation. In the 15 ng/L lineage, brood number remained significantly reduced in F2’, and by F3’, brood number was still numerically lower but not statistically different from controls.

#### 3.2.3. Reproductive Output: Average Offsprings per Brood and Total Offspring per Surviving Female After 21 d (Figure 2C,D)

Reproductive output showed the strongest and most consistent response to Ag^+^ across generations. Both cumulative fecundity (total offspring) and per-brood investment (average offsprings per brood) declined with increasing concentrations, and the effect sizes among treatments increased with successive exposure generations.

Offspring number per brood (Figure 2C): The average number of offspring per brood decreased with increasing concentration and with successive exposure generations. While F0 showed limited separation among the treatment groups, reductions became significant and much clearer from F1 onward. By F2 and especially F3, the 15 ng/L group was significantly lower than the control and lower-concentration groups (0.05, 0.5, and 5 ng/L). In F3, the 5 ng/L group was also significantly lower than the control and the 0.05 and 0.5 ng/L groups. The 0.5 ng/L group likewise showed a reduction at the end of F3 relative to the control and the 50 pg/L group, whereas the control and 50 pg/L groups did not differ significantly throughout the experiment. During recovery, the 0.5 and 5 ng/L lineages showed transient reductions in F1’–F2’ but returned to control levels by F3’, whereas the 15 ng/L lineage remained significantly decreased across F1’–F3’.

Total offspring per surviving female (21 d) (Figure 2D): Total offspring decreased with increasing Ag^+^ concentration, and the effect size increased with ongoing exposure of successive generations. Significant reductions occurred at 5 and 15 ng/L from F0 onward. The 500 pg/L group also showed significantly reduced offspring numbers in F2 and F3, and even 50 pg/L significantly reduced the offspring numbers in F2. By the end of four consecutive exposure generations (F3), total offspring was substantially reduced in the 0.5 and 5 ng/L groups to about 80% and 55% of the control level, respectively. In the 15 ng/L group, the offspring number was reduced to about one third of the control level only. During recovery, the 15 ng/L lineage remained significantly impaired through F3’, whereas the 0.5 and 5 ng/L lineages returned to control levels within two to three recovery generations.

#### 3.2.4. Body Length (Figure 2E)

Body length declined with increasing concentration and with successive exposure generations, with the largest effect size in F3. During the exposure period, the animals in the two highest concentration groups (5 and especially 15 ng/L) were consistently shorter than the controls. Importantly, by F3, even the individuals of two lowest concentration groups (500 and 50 pg/L) were significantly shorter than controls, indicating growth impairment by Ag^+^ at environmentally relevant low concentrations after consecutive multigenerational exposure. During recovery, only the 15 ng/L lineage remained significantly shorter through F3′, while all other lineages were close to control size by the end of the recovery phase. Notably, these low-concentration effects became significant only after consecutive exposure generations, indicating a time-lagged cumulative response that would likely be missed in standard single-generation tests.

#### 3.2.5. Population Growth Rate: Intrinsic Rate of Natural Increase (r) (Figure 2F)

Across treatments, *r* declined with increasing concentration and with successive generations during the exposure phase despite minimal effects on survival, indicating that population-level consequences were driven mainly by changes in reproductive timing and output. In F0, there was little difference in *r* among groups. By F1–F2, the 15 ng/L group shifted to a significantly lower level, and by F3, *r* showed the strongest reduction. From F2 onward, the population growth rate of the 5 ng/L group also became significantly lower than *r* in the controls and continued to decline with the next generation exposed. Importantly, by F3, *r* in the 0.5 ng/L group and the lowest concentration group (0.05 ng/L) was also significantly lower than controls, indicating measurable population-level effects even at picogram-per-liter concentrations after consecutive multigenerational exposure. During recovery, *r* in the 0.05 and 0.5 ng/L lineages returned to the control level after the first recovery generation (F1’), and the 5 ng/L lineage returned to the control level within two recovery generations (F2’). In contrast, *r* remained significantly suppressed in the 15 ng/L lineage through F3′. This pattern underscores that population-level effects emerged progressively across generations rather than in the first exposed generation.

### 3.3. Results for Experimental Series 2: Toxicity and Recovery Dynamics Across Generations (F0–F3) After Different Exposure Histories at 15 ng/L Ag^+^

This experimental series represents different exposure-history scenarios at 15 ng/L Ag^+^ across generations, including continuous exposure, early transfer to clean medium, late transfer to clean medium, and intermittent patterns. The aim was to determine whether outcomes and recovery depend more on exposure timing or on cumulative exposure across generations. This experiment compared exposure-history patterns derived from the 15 ng/L lineage across four generations (F0–F3).

#### 3.3.1. Time to First Reproduction (Figure 3)

Time to first reproduction differed among exposure histories and was best explained by the cumulative number of exposure generations, rather than the specific order of exposed versus clean generations. The fully continuous exposure history (15-15-15-15) showed progressive delays from F1 to F3 and had the latest timing. The lineage exposed only in F0 recovered by F2. In F2, animals with two-generation exposure history (15-15-C) were reproducing significantly later than the controls (C-C-C). In F3, all histories with three exposed generations (15-15-15-C, 15-15-C-15, and 15-C-15-15) extended the time to first reproduction significantly compared to controls (C-C-C-C). In all generations, the time to first reproduction depended primarily on the total number of exposure generations.

**Figure 3:**
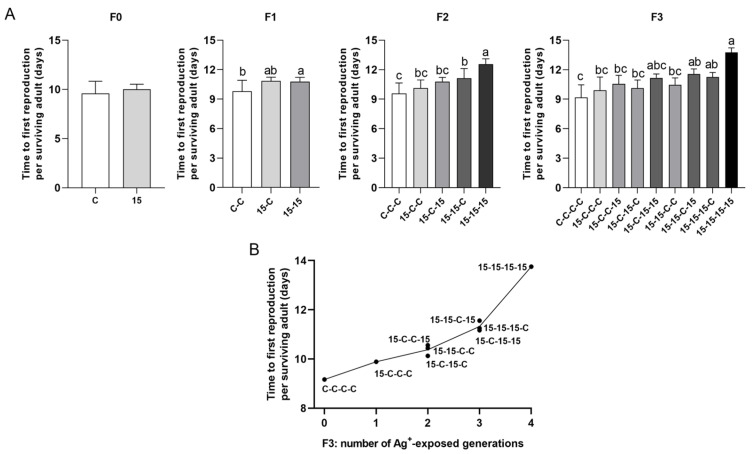
Time to first reproduction in *Daphnia magna* under different 15 ng/L Ag^+^ exposure histories (experimental series 2). (**A**) Time to first reproduction per surviving adult (means + SD) across generations F0–F3 for 15 ng/L exposure histories and the corresponding control histories. Exposure histories are coded as up-to-four-character sequences. (**B**) Mean time to first reproduction of *D. magna* in F3 vs. number of generations exposed to 15 ng/L Ag^+^ (0–4), independent of exposure history; each point corresponds to a single exposure history. For F0, differences between 15 ng/L and control were tested by Mann–Whitney U test. For F1–F3, differences among exposure histories were analyzed by Kruskal–Wallis test, followed, when significant, by Bonferroni-corrected pairwise comparisons. Sample sizes (n) in (**A**) correspond to the number of surviving adults per treatment and generation (see Supplementary Table S2). Different letters above bars indicate significant differences among exposure histories within a given generation (*p* < 0.05).

#### 3.3.2. Total Number of Broods (Figure 4)

Brood number exhibited weaker and less consistent separation among exposure histories than the time taken to reach first reproduction. Nevertheless, clear trends were shown also for this parameter. After the first exposed generation, the 15 ng/L group produced significantly fewer broods than controls. In F1 and F2, exposure histories did not differ significantly, although continuous exposure histories tended to perform worst. In F3, the control animals (C-C-C-C) generally produced most broods, while some of the histories with longer cumulative exposure (15-C-15-C, 15-15-C-15, and 15-15-15-15) resulted in significantly fewer broods than the control.

**Figure 4:**
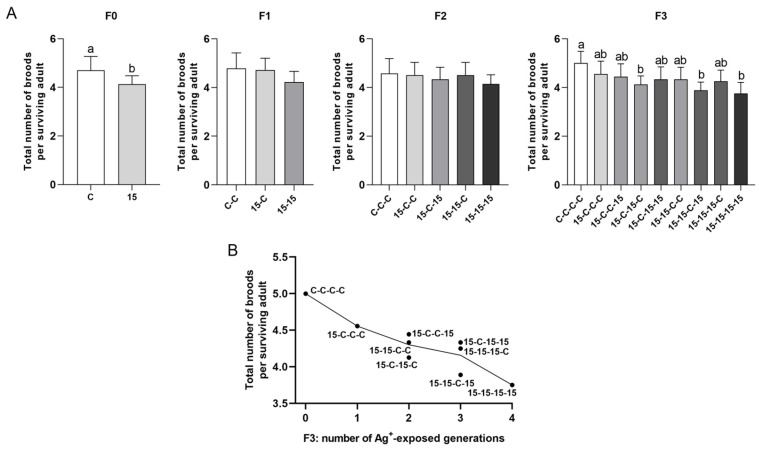
Total number of broods in *Daphnia magna* under different 15 ng/L Ag^+^ exposure histories (experimental series 2). (**A**) Total number of broods per surviving adult (means + SD) across generations F0–F3 for 15 ng/L exposure histories and the corresponding control histories. Exposure histories are coded as in Figure 3. (**B**) Mean total number of broods per surviving adult in F3 vs. number of generations exposed to 15 ng/L Ag^+^ (0–4), independent of exposure history; each point corresponds to a single exposure history. For F0, differences between 15 ng/L and control were tested by Mann–Whitney U test. For F1–F3, differences among exposure histories were analyzed by Kruskal–Wallis test, followed, when significant, by Bonferroni-corrected pairwise comparisons. Sample sizes (n) in (**A**) correspond to the number of surviving adults per treatment and generation (see Supplementary Table S2). Different letters above bars indicate significant differences among exposure histories within a given generation (*p* < 0.05).

#### 3.3.3. Reproductive Output: Total Offspring per Surviving Female (21 d) and Average Offsprings per Brood (Figure 5 and Figure 6)

**Figure 5:**
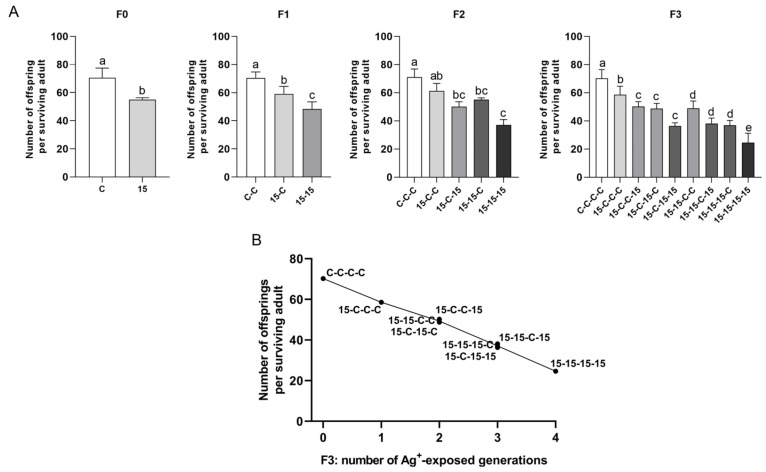
Total number of offspring in *Daphnia magna* under different 15 ng/L Ag^+^ exposure histories (experimental series 2). (**A**) Total number of offspring per surviving adult over 21 d (means + SD) across generations F0–F3 for 15 ng/L exposure histories and the corresponding control histories. Exposure histories are coded as in Figure 3. (**B**) Mean total number of offspring per surviving adult in F3 vs. number of generations exposed to 15 ng/L Ag^+^ (0–4), independent of exposure history; each point corresponds to a single exposure history. For F0, differences between 15 ng/L and control were tested by Mann–Whitney U test. For F1 and F3, differences among exposure histories were analyzed by one-way ANOVA, followed, when significant, by LSD multiple comparisons. For F2, differences among exposure histories were analyzed by Kruskal–Wallis test, followed, when significant, by Bonferroni-corrected pairwise comparisons. Sample sizes (n) in (**A**) correspond to the number of surviving adults per treatment and generation (see Supplementary Table S2). Different letters above bars indicate significant differences among exposure histories within a given generation (*p* < 0.05).

**Figure 6:**
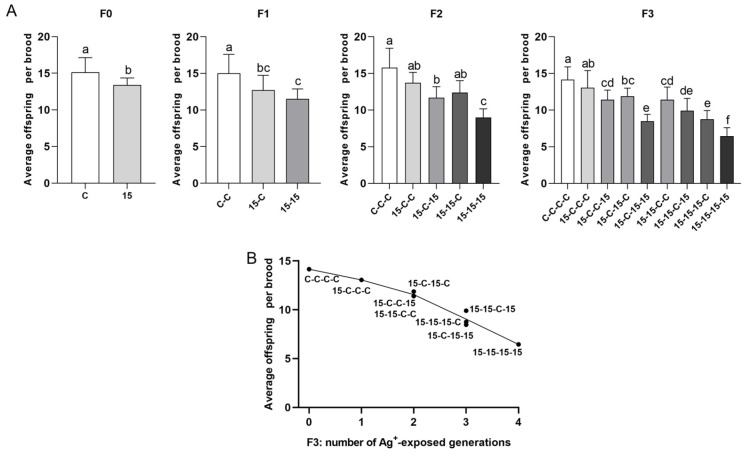
Average offspring per brood in *Daphnia magna* under different 15 ng/L Ag^+^ exposure histories (experimental series 2). (**A**) Average offspring per brood (means + SD) across generations F0–F3 for 15 ng/L exposure histories and the corresponding control histories. Exposure histories are coded as in Figure 3. (**B**) Mean average offspring per brood in F3 vs. number of generations exposed to 15 ng/L Ag^+^ (0–4), independent of exposure history; each point corresponds to a single exposure history. For F0, differences between 15 ng/L and control were tested by Student’s *t*-test. For F1 and F3, differences among exposure histories were analyzed by one-way ANOVA, followed, when significant, by LSD multiple comparisons. For F2, differences among exposure histories were analyzed by Kruskal–Wallis test, followed, when significant, by Bonferroni-corrected pairwise comparisons. Sample sizes (n) in (**A**) correspond to the number of surviving adults per treatment and generation (see Supplementary Table S2). Different letters above bars indicate significant differences among exposure histories within a given generation (*p* < 0.05).

Results obtained for both reproductive output parameters resulted in the clearest ranking among exposure histories and consistently declined as cumulative number of exposed generations increased. Regarding the total offspring per surviving female (21 d) (Figure 5), controls had the highest offspring output in each generation (C, C-C, C-C-C, and C-C-C-C), whereas the fully continuous exposure history resulted in the lowest output in each generation (15, 15-15, 15-15-15, and 15-15-15-15). Mixed histories fell between these extremes, and the offspring counts clustered in respect to the number of exposed generations. Regarding the average offspring number per brood (Figure 6), controls consistently produced the highest average number of offspring per brood, and the fully continuous exposure history fell consistently lowest. Mixed histories aligned in intermediate ranked groups.

By F3 (Figure 5B and Figure 6B), histories with three Ag^+^-exposed generations (15-15-15-C, 15-15-C-15, and 15-C-15-15) clustered within the same homogeneous subset despite different exposure sequences. Two-generation exposure histories (15-15-C-C, 15-C-15-C, and 15-C-C-15) were statistically indistinguishable. The one-generation exposure history (15-C-C-C) consistently showed higher performance than histories with two, three, or four exposed generations. Controls had the highest offspring values, and daphnids continuously exposed over four generations had the lowest.

#### 3.3.4. Body Length (Figure 7)

Body length also followed the exposure history, with smaller size associated with longer cumulative exposure across generations. The control groups (C, C-C, C-C-C, C-C-C-C) always comprised the largest individuals, whereas the 15-15-15-15 animals were smallest. Mixed histories were intermediate.

**Figure 7:**
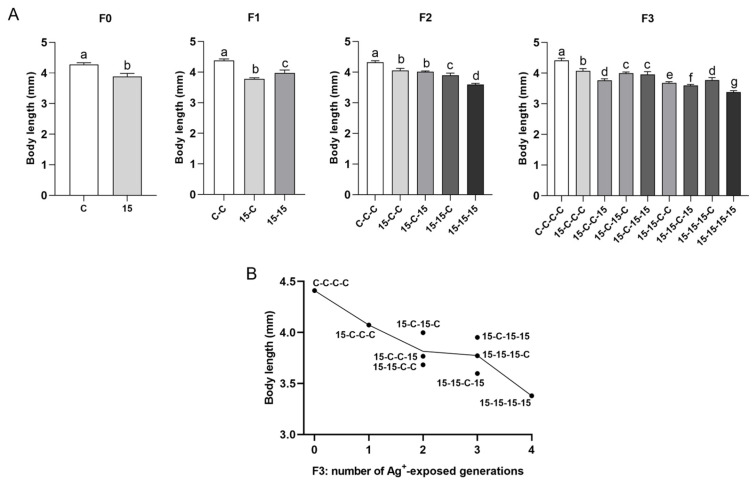
Body length of *Daphnia magna* under different 15 ng/L Ag^+^ exposure histories (experimental series 2). (**A**) Body length (mm; means + SD) at the end of the 21-d test across generations F0–F3 for 15 ng/L exposure histories and the corresponding control histories. Exposure histories are coded as in Figure 3. (**B**) Mean body length of *D. magna* in F3 vs. number of generations exposed to 15 ng/L Ag^+^ (0–4), independent of exposure history; each point corresponds to a single exposure history. For F0, differences between 15 ng/L and control were tested by Student’s *t*-test. For F1–F3, differences among exposure histories were analyzed by one-way ANOVA, followed, when significant, by LSD multiple comparisons. Sample sizes (n) in (**A**) correspond to the number of surviving adults per treatment and generation (see Supplementary Table S2). Different letters above bars indicate significant differences among exposure histories within a given generation (*p* < 0.05).

#### 3.3.5. Population Growth Rate: Intrinsic Rate of Natural Increase (r) (Figure 8)

Data obtained for the parameter *r* showed a clear ranking across exposure histories that matched the ranking for the reproductive output data. The control history (C-C-C-C) had the highest *r*, the fully continuous exposure history (15-15-15-15) had the lowest, and mixed histories were intermediate. Histories with the same number of exposure generations tended to cluster regardless of exposure sequence.

**Figure 8:**
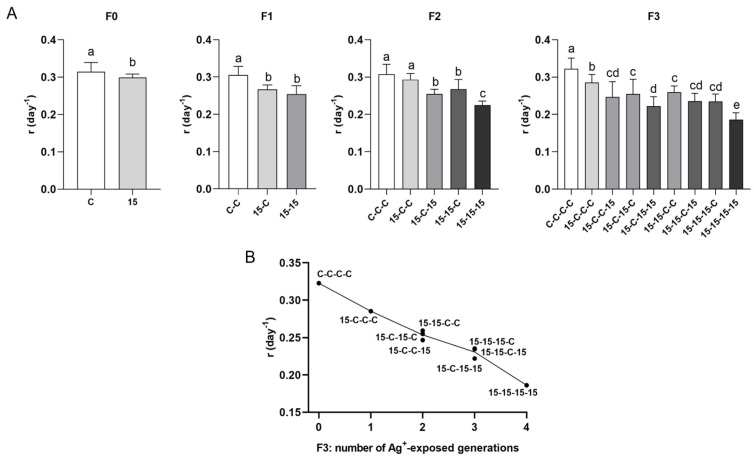
Intrinsic rate of natural increase (*r*) in *Daphnia magna* under different 15 ng/L Ag^+^ exposure histories (experimental series 2). (**A**) Intrinsic rate of natural increase (*r*, day^−1^; means + SD) across generations F0–F3 for 15 ng/L exposure histories and the corresponding control histories. Exposure histories are coded as in Figure 3. (**B**) Mean *r* in F3 *vs*. number of generations exposed to 15 ng/L Ag^+^ (0–4), independent of exposure history; each point corresponds to a single exposure history. For F0, differences between 15 ng/L and control were tested by Student’s *t*-test. For F1–F3, differences among exposure histories were analyzed by one-way ANOVA, followed, when significant, by LSD multiple comparisons. For *r*, n includes all individuals (surviving and dead), giving n = 20 for controls and n = 10 for each Ag^+^ exposure history (see Supplementary Table S2). Different letters above bars indicate significant differences among exposure histories within a given generation (*p* < 0.05).

### 3.4. Results for Experimental Series 3: Transgenerational Effects (15-C-C-C vs. 15-15-15-15-C-C-C) After Short and Long Exposure Histories

This experimental series included two contrasting contamination histories followed by the same recovery period: a single-generation exposure pulse versus prolonged multigenerational exposure, both followed by three generations in clean medium. The aim was to test whether prolonged exposure produces stronger and more persistent transgenerational carryover effects than a short exposure history under identical recovery conditions. Two exposure histories could be directly compared: the continuous four-generation exposure followed by three-generation recovery (15-15-15-15-C-C-C) and the single-generation exposure followed by three-generation recovery (15-C-C-C).

#### 3.4.1. Time to First Reproduction (Figure 9A)

**Figure 9:**
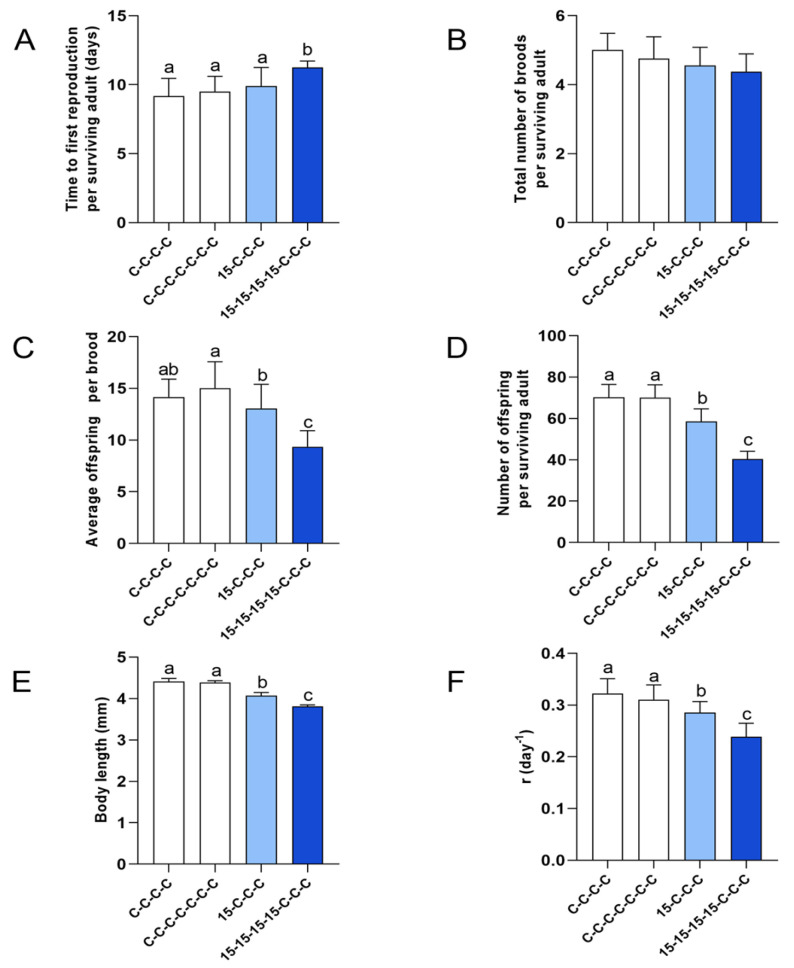
Comparison of short *versus* prolonged 15 ng/L Ag^+^ exposure history followed by three recovery generations (experimental series 3). Life-history traits and intrinsic rate of natural increase (mean + SD) in F3’ for four exposure histories: control lineage over four generations (C-C-C-C), extended control lineage over seven generations (C-C-C-C-C-C-C), a single-generation exposure followed by three recovery generations (15-C-C-C, light blue), and a four-generation exposure followed by three recovery generations (15-15-15-15-C-C-C, dark blue). (**A**) Time to first reproduction (days). (**B**) Total number of broods. (**C**) Average offspring per brood. (**D**) Total number of offspring per surviving adult over 21 d. (**E**) Body length (mm). (**F**) Intrinsic rate of natural increase (*r*, day^−1^). For (**A**–**E**), sample sizes (n) correspond to the number of surviving adults per treatment (see Supplementary Tables S1 and S2). For (**F**), n includes all individuals (surviving and dead), giving n = 20 for control lineages and n = 10 for each 15 ng/L exposure history. Differences among exposure histories in (**A**,**B**) were analyzed by Kruskal–Wallis test, followed, when significant, by Bonferroni-corrected pairwise comparisons. Differences among exposure histories in (**C**–**F**) were analyzed by one-way ANOVA, followed, when significant, by LSD multiple comparisons. Different letters above bars indicate significant differences among exposure histories (*p* < 0.05).

Time to first reproduction (Figure 9A): Controls showed the earliest time to first reproduction, whereas 15-15-15-15-C-C-C remained the latest during recovery. Time to first reproduction in 15-C-C-C returned to control levels during recovery. Specifically, 15-15-15-15-C-C-C remained significantly delayed relative to controls and to 15-C-C-C across recovery generations (F1′–F3′), while 15-C-C-C was not statistically different from controls by the end of recovery (F3′).

#### 3.4.2. Total Number of Broods (Figure 9B)

Total brood number showed only a trend among exposure histories. Differences were not significant, indicating that stronger exposure-history signals were expressed mainly through timing and reproductive output rather than brood counts.

#### 3.4.3. Reproductive Output: Total Offspring per Surviving Female (21 d) and Average Offsprings per Brood Figure 9C,D)

Controls showed the highest total offspring production and the highest average number of offspring per brood, whereas 15-15-15-15-C-C-C showed the lowest values for both endpoints. Although reproductive output in 15-C-C-C remained significantly lower than controls, it was significantly higher than 15-15-15-15-C-C-C. Whereas the time to first reproduction did recover fully after a single-generation pulse of Ag^+^ exposure (15-C-C-C), the offspring number per animal showed partial recovery only and remained below the controls. Multigenerational exposure prior to the recovery period (15-15-15-15-C-C-C) resulted in a significant reduction in offspring numbers, both per brood and per individual.

#### 3.4.4. Body Length (Figure 9E)

Controls had the largest body length, whereas 15-15-15-15-C-C-C animals were smallest. Although the body length in 15-C-C-C remained significantly shorter than controls, it was significantly longer than 15-15-15-15-C-C-C, indicating partial recovery after a single-generation pulse but a persistent reduction in growth after multigenerational exposure prior to the three non-exposed generations.

#### 3.4.5. Population Growth Rate: Intrinsic Rate of Natural Increase (r) (Figure 9F)

Controls exhibited the highest *r*, whereas the 15-15-15-15-C-C-C group showed the lowest. Although *r* for 15-C-C-C remained significantly lower than for controls, it was significantly higher than for 15-15-15-15-C-C-C, indicating that multigenerational exposure history resulted in a larger demographic impairment than a single-generation pulse after the same recovery period.

## 4. Discussion

Our study shows that declines in population-relevant parameters can occur independently of increased mortality, and that they can be caused by even the lowest concentrations of environmental pollutants. In our study, survival remained high across all experimental series and did not explain treatment-related differences in other endpoints. Reproductive timing, reproductive output, body length, and the population growth rate (*r*) generally showed stronger impairment with increasing concentration and/or increasing cumulative number of exposed generations. In the 15 ng/L exposure-history series, multiple endpoints aligned more closely with the cumulative number of exposure generations than with the order of exposed and clean generations. In the concentration-gradient series, significant effects emerged at environmentally relevant low concentration after consecutive exposure, including reduced body length and reduced *r* at 50 pg/L (nominal LOEC) in F3. Recovery depended on concentration and exposure history, with low-concentration lineages typically returning to control levels within one to three recovery generations, whereas the 15 ng/L lineage showed the most persistent impairments after transfer to clean medium. Brood number was generally less sensitive than reproductive timing and output. The targeted comparison further showed that, after the same recovery period, prolonged exposure history produced larger residual deficits than a single-generation pulse.

### 4.1. Low-Concentration Multigenerational Effects and Implications for Environmental Thresholds

In our concentration gradient (experimental series 1), survival remained high across treatments in every generation, suggesting limited lethal toxicity at the tested Ag^+^ concentrations. In contrast, sublethal effects accumulated over time: by F3, body length and the intrinsic rate of natural increase (*r*) were significantly reduced even at 50 pg/L (nominal LOEC), accompanied by delayed time to first reproduction and reduced reproductive output (offspring per brood and per individual). Importantly, this nominal LOEC emerged gradually across generations rather than in the first exposed generation, providing an intuitive demonstration of time-lagged cumulative toxicity that standard acute and short-term single-generation tests [21,46,47,51] are unlikely to detect. This observation aligns with a growing consensus that short-term assays may be inadequate for predicting outcomes in natural ecosystems subject to persistent pollution because they do not capture effects that manifest or amplify across generations [40,59].

The nominal LOEC of 50 pg/L is particularly noteworthy when interpreted in a regulatory context. Because the effective concentration was found to be extremely low, corresponding to 50 parts per quadrillion (ppq) or 5 × 10^−14^. The European Union annual-average environmental quality standard for Ag and its compounds in fresh waters (AA-QS_eco,fw_) is 0.01 µg/L (=10,000 pg/L) and has been endorsed by the Scientific Committee on Health, Environmental, and Emerging Risks [21,56]. Our data show significant multigenerational impairments at concentrations 200-fold below this benchmark, and notably in endpoints that directly integrate population fitness (*r*) and growth. Collectively, these results highlight the advantages of long-term multigenerational study designs in terms of ecological relevance and sensitivity for pollutants subject to persistent environmental exposure, as this approach more accurately reflects real-world population dynamics and reduces uncertainty in long-term risk assessments [43,60,61]. Similar conclusions have been reached across species, where multigenerational experiments often reveal higher sensitivity and improved predictive reliability for chronic field-relevant exposure compared with short-term tests [60,62], which can overlook persistent or progressively worsening impacts within natural populations [34,59].

This cumulative decline in fitness (symbolized by, e.g., reduced reproduction) indicates that the impact of chronic Ag exposure amplifies over time and across generations rather than plateauing. Similar multigenerational deterioration has been reported in other aquatic organisms. For instance, continuous exposure of the midge *Chironomus riparius* to 3 µg/L Ag^+^ caused increased mortality and reduced fertility and population growth rate across four continuous generations [34]. Alongside these and also our present results, multigenerational studies in *Daphnia* and other species confirm that effects often become more pronounced in successive generations, especially under sustained sublethal stress. Notably, using only a single or a few generations can underestimate these chronic effects, as sensitivity may fluctuate until enough generations have passed for responses to stabilize. Indeed, a three-generation study in *D. magna* exposed to the fungicide prochloraz showed clear generation-dependent responses: after F0 exposure, the embryonically exposed F1 offspring showed no significant effects, whereas multiple parameters differed significantly from controls in the potentially germline-exposed F2 generation, indicating a generational time lag in toxicity that single-generation tests could miss [63]. This kind of generation-dependent change in life-history traits is also consistent with multigenerational studies in *D. magna* using other stressors. For example, under thermal stress, *D. magna* showed increased offspring rates in F0 at 25 °C, but the F3 generation had the lowest offspring number and highest oxidative stress, with apparent recovery only after a number of further generations [38]. This reinforces that long-term, multigenerational exposure can detect accumulating impairment that shorter tests might fail to capture. Therefore, our observation of no attenuation in Ag^+^ toxicity over four generations is therefore in line with the expectation that continuous silver ion stress will exert a cumulative multigenerational effect. In line with other publications, this suggests that in both insects and cladocerans, reproductive performance is a vulnerable trait under prolonged low-level contaminant stress. Population growth rate, which integrates fecundity, is consequently depressed over time, as seen in our study and others [34,38,64], underscoring that multigenerational reproductive suppression can threaten long-term population viability. In contrast, traits like development time or adult size may show smaller or transient effects, as organisms can sometimes compensate developmentally even if reproductive capacity continues to decline [34,65,66]. Overall, the pronounced multigenerational decline in the reproductive success of *D. magna* observed here aligns with the broader evidence that chronic pollutants primarily undermine reproductive fitness across generations, a finding of high concern for ecological risk evaluations.

We also examined whether *D. magna* could acclimate or recover after chronic exposure once the stressor was removed, and our results provide insight into the balance between physiological acclimation and transgenerational effects. In our study, the offspring under continuous exposure of the highest test concentration did not fully revert to control performance even after a recovery period of three generations This pattern is consistent with the observations of Ding et al. [34], who found life-cycle parameter impairments in *C. riparius* persisting across at least three unexposed generations following four generations exposed to Ag^+^ at concentrations of 0.1 µg/L or higher. This phenomenon, in principle, has also been noted by other researchers: if organisms fail to fully recover after a contaminant is removed, it implies that heritable changes (e.g., epigenetic modifications), rather than simple reversible acclimation processes, have occurred. Conversely, full recovery in the next generation would indicate that the effects were primarily due to physiological plasticity with no permanent legacy. Also, our findings of sustained reproductive depression and delayed recovery following higher Ag^+^ concentrations imply that the silver-induced damage to a generation has not fully been reset in the offspring, pointing to potential epigenetic or maternal-effect mechanisms. Further biochemical or genomic analyses (e.g., DNA methylation assays) are needed to confirm the exact mechanism in *D. magna*.

### 4.2. Exposure History and the Number of Exposed Generations as the Main Driver

Experimental series 2 was designed to test whether, at 15 ng/L Ag^+^, the pattern of exposure across generations (early vs. late exposure, and continuous vs. intermittent) matters as much as the total number of exposed generations. Consistent patterns across the parameters time to first reproduction, average offspring per brood, total offspring per individual, body length, and *r* emerged: lineages with the same number of exposed generations tended to show similar responses, regardless of which specific generations were exposed. At 15 ng/L, lineages with three or four exposed generations consistently showed the latest reproduction start, the smallest body size, the lowest average brood size and total offspring number, and the lowest *r*, while lineages with only one or two exposed generations revealed intermediate impairment. This pattern indicates that the total number of exposed generations was more important than the exact order of exposed vs. clean generations for determining long-term life-history and population-growth outcomes in *D. magna* and thus suggests that epigenetic modifications that are stable over several generations have been induced by exposure to silver ions.

A similar logic emerges from multigenerational Ag nanoparticle (AgNP) and Ag^+^ studies in other taxa. In the nematode *Caenorhabditis elegans* continuous multigenerational exposure to Ag^+^ and AgNPs produced elevated reproductive sensitivity and persistent effects even after four clean generations, highlighting the importance of exposure-history length [31]. In *C. riparius*, Ding et al. [34] combined a pulsed high-concentration exposure of the aquatic larvae with intervening recovery periods. They demonstrated that cumulative exposure can drive persistent reductions in life-cycle parameters and population growth, with recovery depending on how many generations were exposed. Our results show that this exposure-history dependence also appears at extremely low Ag^+^ concentrations in *D. magna*, reinforcing the need to treat the number of exposed generations as an explicit factor in ecological risk assessment.

These findings have two important implications. First, they show that intermittent contamination, if it recurs across generations, can still produce strong effects, even if exposure events in the lineage are separated by clean intervals. Second, they indicate that reducing the cumulative number of exposed generations is beneficial: lineages with only one or two exposed generations generally showed better life-history and population-growth responses than those with three or four exposed generations at the same concentration.

### 4.3. Persistence After Removal: Different Recovery After Short vs. Prolonged Exposure

Experimental series 3 contrasted short (15-C-C-C) and prolonged (15-15-15-15-C-C-C) exposure histories, both followed by three generations in clean medium, to examine how recovery potential depends on prior exposure history.

For time to first reproduction, the animals subjected to the one-generation pulse history (15-C-C-C) recovered to the control level within three clean generations, whereas the lineage with prolonged exposure history (15-15-15-15-C-C-C) remained delayed. This finding suggests putative non-genetic modifications to occur that can be reversed relatively quickly after a single pulse but take much more time and generations to normalize after prolonged multigenerational exposure.

For the reproductive output and *r*, recovery was even more limited. Total offspring and average offspring per brood remained significantly lower than controls in both histories, but the prolonged exposure history (15-15-15-15-C-C-C) resulted in a stronger and more persistent reduction than 15-C-C-C. Similarly, *r* was highest in control lineages, lowest in 15-15-15-15-C-C-C, and intermediate in 15-C-C-C, with the latter still significantly below the control level despite partial recovery. This pattern suggests that different life-history components recover at different rates, and that prolonged exposure can leave severe epigenetic or physiological modifications, resulting in long-lasting deficits in reproductive investment and population growth that are not fully erased by three generations in clean medium even though the last ‘recovered’ generation was never exposed to Ag^+^, not even in the germline of the mother organism’s embryos.

Such incomplete recovery is consistent with other multigenerational studies showing that, even after exposure cessation, reproduction and overall population performance can remain altered. In *D. magna*, multigenerational exposure to AgNPs has been reported to produce persistent effects on reproduction and body size across subsequent generations, alongside molecular responses interpreted as epigenetic carryover, with the extent of recovery depending on exposure duration and the nanoparticle aging state [50]. By contrast, a separate six-generation study demonstrated that these long-term reproductive effects were prominent for pristine AgNPs but were largely absent for wastewater-borne (transformed) AgNPs, underscoring how environmental transformation can strongly modulate multigenerational toxicity [49]. In *C. elegans*, six generations of Ag^+^ exposure likewise led to sustained increases in reproductive sensitivity that persisted through four recovery generations, with post-recovery sensitivity remaining closer to that of the last exposed generation than to unexposed controls [31]. Together, these findings support our observation that Ag^+^ can produce long-lasting, exposure-history-dependent effects in *D. magna* even at extremely low concentrations. Several mechanistic pathways could underline the multi- and transgenerational effects observed.

The following sub-organismic physiological and molecular effects of Ag^+^ can serve as an explanation for the observed effects at the population level. At the physiological level, Ag^+^ is known to interfere with ion regulation in *D. magna*, particularly by inhibiting Na^+^ uptake and Na^+^/K^+^-ATPase activity, which can disturb osmoregulation and the acid–base balance [67]. Such disturbances increase the energetic costs of homeostasis and can reduce the energy available for growth and reproduction, providing a plausible explanation for the simultaneously occurring phenomena of smaller body size, delayed reproduction, and reduced reproductive output in our Ag^+^-exposed lineages. Biological mediation, especially via the gut microbiome, may also contribute. Li et al. [68] showed that, during four generations of exposure in *D. magna*, gut-microbial adaptation can mediate detoxification by transforming Ag species (Ag^+^ and AgNPs), with generational shifts in microbial community structure and silver speciation associated with reduced toxic symptoms. Other multigenerational *Daphnia* studies in complex environmental matrices likewise link long-term responses to coordinated changes across organismal performance and molecular profiles, including global DNA methylation and proteomics [69,70], while recent work demonstrates that proteome-level changes can accompany gradual multigenerational acclimation to organic contaminants [71]. These findings suggest that microbial and molecular adjustments could support the slow and incomplete recovery observed after prolonged Ag^+^ exposure, even when external concentrations have returned to zero. In *D. magna*, exposure to Ag has also been linked to impaired reproductive investment due to disrupted oogenesis and vitellogenin provisioning [36], as well as to internalization and tissue-level responses following exposure to various forms of Ag [48,51,72]. This provides additional mechanistic context for the consistent multigenerational reductions in growth and reproduction observed in the present study.

Our results complement and extend previous multigenerational studies on Ag toxicity in several ways. First, most multigenerational work with *D. magna* has focused on nanoparticulate silver and often in µg/L concentrations [30,49,50,73]. In contrast, we examined Ag^+^ in the pg/L range, closely aligned with and far below current and proposed environmental thresholds. We show that the population growth rate (*r*) can be significantly reduced at 50 pg/L after repeated exposure, even though the survival remained unaffected. This underscores the importance of including integrated life-history endpoints such as *r* in toxicity assessment, which combines reproduction timing and fecundity, being more sensitive to multigenerational exposure than survival alone [38].

Second, by embedding three experimental series into one framework, our study provides a direct quantitative comparison of concentration-gradient effects with recovery, different exposure-history patterns at 15 ng/L, and short versus prolonged exposure history followed by identical recovery. The consistent finding that the number of exposed generations is more important than their order simplifies how long-term exposure histories can be conceptualized in risk assessment: a ‘cumulative exposed generations’ metric captures much of the relevant biological response.

Third, the patterns observed here in *D. magna* are consistent with multigenerational Ag effects observed in other studies conducted with *D. magna* and many other invertebrates. For example, multigenerational AgNP exposure on *D. magna* has been shown to result in effects that carry over into unexposed recovery generations, indicating incomplete recovery after exposure cessation [50]. Multigenerational exposure to Ag^+^ and AgNPs in the nematode *C. elegans* resulted in elevated reproductive sensitivity, with persistent effects even when exposure was interrupted after four generations [31]. Long-term Ag^+^ exposures in *C. riparius* at low ng/L levels also showed that life-cycle parameters and population growth can be impaired across multiple generations, with incomplete recovery after non-exposed life stages (i.e., adults) and even three recovery generations [34]. Taken together, these studies and our results indicate that silver, whether present as ions or as transformed nanoparticles, can induce multigeneration changes in life-history traits and population growth in invertebrates at low concentrations.

Ecotoxicological risk assessment has conventionally used short-term toxicity endpoints generated under controlled laboratory conditions and extrapolated these to predicted no-effect concentrations to provide long-term protection of field populations. In several jurisdictions, this extrapolation incorporates assessment factors to address uncertainties when translating laboratory effect concentrations to environmental scenarios, though such factors are not always applied. The selected factors can vary from highly conservative (division by 1000) to relatively low (division by 3) for endpoints including ECx and no-observed-effect concentrations (NOECs) [74,75]. There is no exception for *D. magna* either. The standard *D. magna* 21-day reproduction test [27] is widely used for deriving chronic NOECs for regulatory purposes, but by design, it evaluates only a single generation. Our data show that multigenerational exposure to a series of low Ag^+^ concentrations can (1) sharpen differences among these concentrations, (2) reveal significant effects at very low concentrations (e.g., 0.05 ng/L) right after several exposed generations, and (3) show that recovery depends on how many generations have been previously exposed. From a regulatory perspective, the observed phenomenon of Ag^+^-exposed *D. magna* in our study challenges the reliability of traditional short-term experiments and assessment factor-based approaches. Because the data may not be sufficiently protective when contamination is chronic or intermittent, especially when the contaminant can persist in sediments, biota, or infrastructure and repeatedly affect successive generations. Consistent with our findings, the >10-fold increase in sensitivity observed when comparing parental generation nematodes *C. elegans* with cohorts exposed multigenerationally to Ag-PVP and AgNO_3_ calls into question the validity of this assessment factor-based strategy [31]. Multigenerational exposure alone explains an approximately one-order-of-magnitude divergence between effects measured in short- versus long-term exposures, indicating that environmental risk may be substantially higher than that inferred from short-term tests [31]. Incorporating multigenerational life-history endpoints and the intrinsic rate of natural increase (*r*, representing population growth) into hazard characterization could reveal additional risks at concentrations that appear safe in standard 21-day tests. Reviews and multigeneration *Daphnia* studies emphasize that long-term tests often detect changes in reproduction, growth, and *r* that are not evident in single-generation exposures, and that both adverse and compensatory responses can appear depending on the stressor and exposure pattern [37,38,76,77]. Our findings for Ag^+^ in *D. magna* fit into this body of evidence and support the use of multigeneration designs in situations where contamination is expected to be long-term or recurrent.

This study has several considerations that are relevant for interpreting the results and for future work. First, all Ag^+^ concentrations are nominal rather than analytically measured. It should be noted that the chloride content and the alkalinity provided by the major salts used to prepare ISO medium [53] can shift Ag away from the free-ion pool through complexation and/or formation of less bioavailable phases, thereby lowering free Ag^+^ relative to nominal dissolved concentrations. Accordingly, the actual free Ag^+^ concentrations experienced by the *D. magna* may have been even lower than the nominal values for at least part of each renewal interval. This implies that the observed multigenerational LOEC (nominal 50 pg/L) reflects effects that can manifest even under chemically buffered conditions, strengthening the interpretation of our results as conservative (worst-case) hazard estimates under long-term exposure scenarios. In particular, in natural waters, dissolved silver speciation is strongly influenced by precipitation, redox, or photochemical processes and complexation with chloride, sulfide, and dissolved organic matter, which the bioavailable Ag^+^ fraction may be further reduced, and effect magnitudes may differ among sites [45,78,79]. Thus, our use of a well-defined ionic Ag^+^ concentration series in ISO medium likely represents a relative conservative (worst-case) scenario relative to many natural systems, strengthening the argument that if effects are observed under such controlled conditions, they should not be ignored when defining environmental thresholds.

Future work could test whether persistent changes in life-history traits and *r* are linked to multigenerational oxidative-stress dynamics (e.g., ROS production, lipid peroxidation, and antioxidant defenses), which have been shown to vary across generations in *D. magna* under thermal or chemical stress [37,38,80] and in other invertebrates under silver exposure [32]. It would also be valuable to examine microbiome composition and gut-mediated metal transformation [68], and specific epigenetic signatures, including DNA methylation and chromatin-associated marks such as histone modifications, given evidence for pollutant-associated shifts in global DNA methylation in *D. magna* and related systems and the broader relevance of epigenetic memory under metal exposure [69,81,82]. Finally, our results show that for *D. magna*, the number of exposed generations is a key factor controlling long-term outcomes at ng/L Ag^+^. Future multigeneration studies with this species and others could systematically vary both exposure concentration and number of exposed generations, while measuring both life-history traits and mechanistic biomarkers. These studies would support more refined chronic testing strategies and help evaluate whether current silver thresholds are sufficiently protective under long-term exposure conditions.

## Data Availability

The original contributions presented in this study are included in the article/Supplementary Materials. Further inquiries can be directed to the corresponding author.

## Supplementary Materials

The following supporting information can be downloaded at https://www.mdpi.com/article/10.3390/jox16020060/s1, Table S1: Survival rates of *D. magna* in experimental series 1. Table S2: Survival rates of *D. magna* in experimental series 2.

## References

[1] Purcell T.W., Peters J.J. (1998). Sources of silver in the environment. Environ. Toxicol. Chem..

[2] Diamond J.M., Mackler D.G., Collins M., Gruber D. (1990). Derivation of a freshwater silver criteria for the New River, Virginia, using representative species. Environ. Toxicol. Chem..

[3] Wood C.M., Hogstrand C., Galvez F., Munger R. (1996). The physiology of waterborne silver toxicity in freshwater rainbow trout (*Oncorhynchus mykiss*) 1. The effects of ionic Ag^+^. Aquat. Toxicol..

[4] Hiriart-Baer V.P., Fortin C., Lee D.-Y., Campbell P.G. (2006). Toxicity of silver to two freshwater algae, *Chlamydomonas reinhardtii* and *Pseudokirchneriella subcapitata*, grown under continuous culture conditions: Influence of thiosulphate. Aquat. Toxicol..

[5] Kolts J.M., Boese C.J., Meyer J.S. (2009). Effects of dietborne copper and silver on reproduction by *Ceriodaphnia dubia*. Environ. Toxicol. Chem..

[6] Taylor C., Matzke M., Kroll A., Read D.S., Svendsen C., Crossley A. (2016). Toxic interactions of different silver forms with freshwater green algae and cyanobacteria and their effects on mechanistic endpoints and the production of extracellular polymeric substances. Environ. Sci. Nano.

[7] Luoma S.N. (2008). Silver nanotechnologies and the environment. Project Emerg. Nanotechnol. Rep..

[8] Hogstrand C., Wood C.M. (1998). Toward a better understanding of the bioavailability, physiology, and toxicity of silver in fish: Implications for water quality criteria. Environ. Toxicol. Chem..

[9] Hamad A., Khashan K.S., Hadi A. (2020). Silver nanoparticles and silver ions as potential antibacterial agents. J. Inorg. Organomet. Polym. Mater..

[10] Deshmukh S.P., Patil S., Mullani S., Delekar S. (2019). Silver nanoparticles as an effective disinfectant: A review. Mater. Sci. Eng. C.

[11] Künniger T., Gerecke A.C., Ulrich A., Huch A., Vonbank R., Heeb M., Wichser A., Haag R., Kunz P., Faller M. (2014). Release and environmental impact of silver nanoparticles and conventional organic biocides from coated wooden façades. Environ. Pollut..

[12] Funck J.A., Danger M., Gismondi E., Cossu-Leguille C., Guérold F., Felten V. (2013). Behavioural and physiological responses of *Gammarus fossarum* (Crustacea Amphipoda) exposed to silver. Aquat. Toxicol..

[13] Jeremiah S.S., Miyakawa K., Morita T., Yamaoka Y., Ryo A. (2020). Potent antiviral effect of silver nanoparticles on SARS-CoV-2. Biochem. Biophys. Res. Commun..

[14] Hiemstra A.-F., Rambonnet L., Gravendeel B., Schilthuizen M. (2021). The effects of COVID-19 litter on animal life. Anim. Biol..

[15] European Commission Regulation on the Supply and Use of Biocidal Products. Directorate-General for Health and Food Safety. https://health.ec.europa.eu/biocides/regulation_en.

[16] Pereira-Silva P., Borges J., Sampaio P. (2025). Recent advances in metal-based antimicrobial coatings. Adv. Colloid Interface Sci..

[17] Bakar N.H.A., Ismail W.N.W., Umair M., Kumar A.S.K. (2025). Antibacterial Textile Coatings with Strategies for Long-Term Performance and Environmental Safety. Adv. Nanocomposites.

[18] Ratte H.T. (1999). Bioaccumulation and toxicity of silver compounds: A review. Environ. Toxicol. Chem..

[19] Ndungu K. (2011). Dissolved silver in the Baltic Sea. Environ. Res..

[20] Mertens J., Oorts K., Leverett D., Arijs K. (2019). Effects of silver nitrate are a conservative estimate for the effects of silver nanoparticles on algae growth and *Daphnia magna* reproduction. Environ. Toxicol. Chem..

[21] Arijs K., Nys C., Van Sprang P., De Schamphelaere K., Mertens J. (2021). Setting a protective threshold value for silver toward freshwater organisms. Environ. Toxicol. Chem..

[22] Anzecc A. (2000). Australian and New Zealand Guidelines for Fresh and Marine Water Quality.

[23] Du J., Tang J., Xu S., Ge J., Dong Y., Li H., Jin M. (2018). A review on silver nanoparticles-induced ecotoxicity and the underlying toxicity mechanisms. Regul. Toxicol. Pharmacol..

[24] Li F., Li R., Lu F., Xu L., Gan L., Chu W., Yan M., Gong H. (2023). Adverse effects of silver nanoparticles on aquatic plants and zooplankton: A review. Chemosphere.

[25] Rajan R., Huo P., Chandran K., Dakshinamoorthi B.M., Yun S.-I., Liu B. (2022). A review on the toxicity of silver nanoparticles against different biosystems. Chemosphere.

[26] OECD (2004). Test No. 202: Testing of Chemicals: Daphnia sp., Acute Immobilisation Test, Method.

[27] OECD (2012). Test No. 211: Daphnia magna Reproduction Test, OECD Guidelines for the Testing of Chemicals, Section 2.

[28] Reilly K., Ellis L.-J.A., Davoudi H.H., Supian S., Maia M.T., Silva G.H., Guo Z., Martinez D.S.T., Lynch I. (2023). *Daphnia* as a model organism to probe biological responses to nanomaterials—From individual to population effects via adverse outcome pathways. Front. Toxicol..

[29] Boros B.-V., Ostafe V. (2020). Evaluation of ecotoxicology assessment methods of nanomaterials and their effects. Nanomaterials.

[30] Völker C., Boedicker C., Daubenthaler J., Oetken M., Oehlmann J. (2013). Comparative toxicity assessment of nanosilver on three *Daphnia* species in acute, chronic and multi-generation experiments. PLoS ONE.

[31] Schultz C.L., Wamucho A., Tsyusko O.V., Unrine J.M., Crossley A., Svendsen C., Spurgeon D.J. (2016). Multigenerational exposure to silver ions and silver nanoparticles reveals heightened sensitivity and epigenetic memory in *Caenorhabditis elegans*. Proc. R. Soc. B.

[32] Rossbach L.M., Oughton D.H., Maremonti E., Eide D.M., Brede D.A. (2021). Impact of multigenerational exposure to AgNO_3_ or NM300K Ag NPs on antioxidant defense and oxidative stress in *Caenorhabditis elegans*. Ecotoxicol. Environ. Saf..

[33] Wamucho A., Unrine J.M., Kieran T.J., Glenn T.C., Schultz C.L., Farman M., Svendsen C., Spurgeon D.J., Tsyusko O.V. (2019). Genomic mutations after multigenerational exposure of *Caenorhabditis elegans* to pristine and sulfidized silver nanoparticles. Environ. Pollut..

[34] Ding J., Krais S., Li Z., Triebskorn R., Köhler H.-R. (2025). Multi-and Transgenerational Effects of Silver Ions (Ag^+^) in the ng/L Range on Life Cycle Parameters and Population Growth of the Midge *Chironomus riparius* (Diptera, Chironomidae). Toxics.

[35] Lampert W., Kinne O. (2011). Daphnia: Development of a Model Organism in Ecology and Evolution.

[36] Hook S.E., Fisher N.S. (2001). Sublethal effects of silver in zooplankton: Importance of exposure pathways and implications for toxicity testing. Environ. Toxicol. Chem..

[37] Jeong T.-Y., Yuk M.-S., Jeon J., Kim S.D. (2016). Multigenerational effect of perfluorooctane sulfonate (PFOS) on the individual fitness and population growth of *Daphnia magna*. Sci. Total Environ..

[38] Im H., Na J., Jung J. (2020). Multigenerational plasticity of *Daphnia magna* under thermal stress across ten generations. Ecotoxicol. Environ. Saf..

[39] Muyssen B.T., Janssen C.R. (2004). Multi-generation cadmium acclimation and tolerance in *Daphnia magna* Straus. Environ. Pollut..

[40] Araujo G., Abessa D., Soares A., Loureiro S. (2019). Multi-generational exposure to Pb in two monophyletic *Daphnia* species: Individual, functional and population related endpoints. Ecotoxicol. Environ. Saf..

[41] Shi Y., Meng X., Zhang J. (2021). Multi-and trans-generational effects of N-butylpyridium chloride on reproduction, lifespan, and pro/antioxidant status in *Caenorhabditis elegans*. Sci. Total Environ..

[42] Li Z., Yu Z., Gao P., Yin D. (2020). Multigenerational effects of perfluorooctanoic acid on lipid metabolism of *Caenorhabditis elegans* and its potential mechanism. Sci. Total Environ..

[43] Araujo G., Abessa D., Soares A., Loureiro S. (2019). Multi-generational effects under single and pulse exposure scenarios in two monophyletic *Daphnia* species. Sci. Total Environ..

[44] Vandegehuchte M.B., Lemière F., Janssen C. (2009). Quantitative DNA-methylation in *Daphnia magna* and effects of multigeneration Zn exposure. Comp. Biochem. Physiol. C Toxicol. Pharmacol..

[45] Shen M.-H., Zhou X.-X., Yang X.-Y., Chao J.-B., Liu R., Liu J.-F. (2015). Exposure medium: Key in identifying free Ag^+^ as the exclusive species of silver nanoparticles with acute toxicity to *Daphnia magna*. Sci. Rep..

[46] Zhao C.M., Wang W.X. (2011). Comparison of acute and chronic toxicity of silver nanoparticles and silver nitrate to *Daphnia magna*. Environ. Toxicol. Chem..

[47] Ribeiro F., Gallego-Urrea J.A., Jurkschat K., Crossley A., Hassellöv M., Taylor C., Soares A.M., Loureiro S. (2014). Silver nanoparticles and silver nitrate induce high toxicity to *Pseudokirchneriella subcapitata*, *Daphnia magna* and *Danio rerio*. Sci. Total Environ..

[48] Hu Y., Chen X., Yang K., Lin D. (2018). Distinct toxicity of silver nanoparticles and silver nitrate to *Daphnia magna* in M4 medium and surface water. Sci. Total Environ..

[49] Hartmann S., Louch R., Zeumer R., Steinhoff B., Mozhayeva D., Engelhard C., Schönherr H., Schlechtriem C., Witte K. (2019). Comparative multi-generation study on long-term effects of pristine and wastewater-borne silver and titanium dioxide nanoparticles on key lifecycle parameters in *Daphnia magna*. NanoImpact.

[50] Ellis L.-J.A., Valsami-Jones E., Lynch I. (2020). Exposure medium and particle ageing moderate the toxicological effects of nanomaterials to *Daphnia magna* over multiple generations: A case for standard test review?. Environ. Sci. Nano.

[51] Pakrashi S., Tan C., Wang W.X. (2017). Bioaccumulation-based silver nanoparticle toxicity in *Daphnia magna* and maternal impacts. Toxicol. Chem..

[52] Doria H.B., Pfenninger M. (2021). A multigenerational approach can detect early Cd pollution in *Chironomus riparius*. Chemosphere.

[53] (2012). Water Quality—Determination of the Inhibition of the Mobility of *Daphnia magna* Straus (Cladocera, Crustacea)—Acute Toxicity Test.

[54] Fischer L., Smith G., Hann S., Bruland K.W. (2018). Ultra-trace analysis of silver and platinum in seawater by ICP-SFMS after off-line matrix separation and pre-concentration. Mar. Chem..

[55] Ndung’u K., Ranville M.A., Franks R.P., Flegal A.R. (2006). On-line determination of silver in natural waters by inductively-coupled plasma mass spectrometry: Influence of organic matter. Mar. Chem..

[56] Scott M., Vighi M., Borges T., Duarte-Davidson R., Hoet P., de Voogt P., Backhaus T., Johnson A., Linders J. (2021). SCHEER (Scientific Committee on Health, Environmental and Emerging Risks), Final Opinion on “Draft Environmental Quality Standards for Priority Substances Under the Water Framework Directive”, Silver and Its Compounds. http://eprints.imdea-agua.org:13000/1538/1/scheer_o_022.pdf.

[57] Jacobasch C., Volker C., Giebner S., Volker J., Alsenz H., Potouridis T., Heidenreich H., Kayser G., Oehlmann J., Oetken M. (2014). Long-term effects of nanoscaled titanium dioxide on the cladoceran *Daphnia magna* over six generations. Environ. Pollut..

[58] Trotter B., Wilde M.V., Brehm J., Dafni E., Aliu A., Arnold G.J., Fröhlich T., Laforsch C. (2021). Long-term exposure of *Daphnia magna* to polystyrene microplastic (PS-MP) leads to alterations of the proteome, morphology and life-history. Sci. Total Environ..

[59] Im J., Chatterjee N., Choi J. (2019). Genetic, epigenetic, and developmental toxicity of *Chironomus riparius* raised in metal-contaminated field sediments: A multi-generational study with arsenic as a second challenge. Sci. Total Environ..

[60] Tsui M.T., Wang W.X. (2005). Multigenerational acclimation of *Daphnia magna* to mercury: Relationships between biokinetics and toxicity. Environ. Toxicol. Chem..

[61] Segner H., Caroll K., Fenske M., Janssen C., Maack G., Pascoe D., Schäfers C., Vandenbergh G., Watts M., Wenzel A. (2003). Identification of endocrine-disrupting effects in aquatic vertebrates and invertebrates: Report from the European IDEA project. Ecotoxicol. Environ. Saf..

[62] Chen Y., Huang J., Xing L., Liu H., Giesy J.P., Yu H., Zhang X. (2014). Effects of multigenerational exposures of *D. magna* to environmentally relevant concentrations of pentachlorophenol. Sci. Pollut. Res..

[63] Poulsen R., Henrik H., Hansen M., Cedergreen N. (2021). Grandmother’s pesticide exposure revealed bi-generational effects in *Daphnia magna*. Aquat. Toxicol..

[64] Yu Z., Sun G., Liu Y., Yin D., Zhang J. (2017). Trans-generational influences of sulfamethoxazole on lifespan, reproduction and population growth of *Caenorhabditis elegans*. Ecotoxicol. Environ. Saf..

[65] Nguyen N.D., Matsuura T., Kato Y., Watanabe H. (2021). DNMT3. 1 controls trade-offs between growth, reproduction, and life span under starved conditions in *Daphnia magna*. Sci. Rep..

[66] Revel M.L., van Drimmelen C.K., Černíková F., Suarez E.P., Hursthouse A., Heise S. (2026). Toxicity and biodistribution of lanthanum and gadolinium in *Daphnia magna* following chronic dietary and waterborne exposure. Ecotoxicology.

[67] Bianchini A., Wood C.M. (2002). Physiological effects of chronic silver exposure in *Daphnia magna*. Comp. Biochem. Physiol. C Toxicol. Pharmacol..

[68] Li Y., Wang W.-X., Liu H. (2022). Gut-microbial adaptation and transformation of silver nanoparticles mediated the detoxification of *Daphnia magna* and their offspring. Environ. Sci. Nano.

[69] Chatterjee N., Choi S., Kwon O.K., Lee S., Choi J. (2019). Multi-generational impacts of organic contaminated stream water on *Daphnia magna*: A combined proteomics, epigenetics and ecotoxicity approach. Environ. Pollut..

[70] Kim J., Choi J. (2023). Trans-and multigenerational effects of Isothiazolinone biocide CMIT/MIT on genotoxicity and epigenotoxicity in *Daphnia magna*. Toxics.

[71] Boyd A., Lummer C., Mehta D., Uhrig R.G., Blewett T.A. (2025). Getting over it? A proteomic analysis of mechanisms driving multigenerational acclimation to organic ultraviolet filters in *Daphnia magna*. Environ. Pollut..

[72] Scanlan L.D., Reed R.B., Loguinov A.V., Antczak P., Tagmount A., Aloni S., Nowinski D.T., Luong P., Tran C., Karunaratne N. (2013). Silver nanowire exposure results in internalization and toxicity to *Daphnia magna*. ACS Nano.

[73] da Silva M.L.N., Nogueira D.J., Köerich J.S., Vaz V.P., Justino N.M., Schmidt J.R.A., Vicentini D.S., Matias M.S., de Castilhos A.B., Fuzinatto C.F. (2021). Multigenerational toxic effects on *Daphnia magna* induced by silver nanoparticles and glyphosate mixture. Environ. Toxicol. Chem..

[74] Bodar C.W., Pronk M.E., Sijm D.T. (2005). The European Union risk assessment on zinc and zinc compounds: The process and the facts. Integr. Environ. Assess. Manag..

[75] EC (2003). Technical Guidance Document in Support of Commission Directive 93/67/EEC on Risk Assessment for New Notified Substances and Commission Regulation (EC) No 1488/94 on Risk Assessment for Existing Substances.

[76] Suarez E.P., Pugliese S., Galdiero E., Guida M., Libralato G., Saviano L., Spampinato M., Pappalardo C., Siciliano A. (2023). Multigenerational tests on *Daphnia* spp.: A vision and new perspectives. Environ. Pollut..

[77] Giraudo M., Dubé M., Lépine M., Gagnon P., Douville M., Houde M. (2017). Multigenerational effects evaluation of the flame retardant tris (2-butoxyethyl) phosphate (TBOEP) using *Daphnia magna*. Aquat. Toxicol..

[78] Singh A., Hou W.-C., Lin T.-F., Zepp R.G. (2019). Roles of silver–chloride complexations in sunlight-driven formation of silver nanoparticles. Environ. Sci. Technol..

[79] Ben Yahia M., Ben Yahia M. (2020). Physico-chemical study of complexation of silver ion (Ag(^+^)) by macrocyclic molecules (hexa-Helicenes) based on statistical physics theory: New description of a cancer drug. Sci. Rep..

[80] Bae E., Samanta P., Yoo J., Jung J. (2016). Effects of multigenerational exposure to elevated temperature on reproduction, oxidative stress, and Cu toxicity in *Daphnia magna*. Ecotoxicol. Environ. Saf..

[81] Vandegehuchte M.B., De Coninck D., Vandenbrouck T., De Coen W.M., Janssen C.R. (2010). Gene transcription profiles, global DNA methylation and potential transgenerational epigenetic effects related to Zn exposure history in *Daphnia magna*. Environ. Pollut..

[82] Pham K., Ho L., D’Incal C.P., De Cock A., Vanden Berghe W., Goethals P. (2023). Epigenetic analytical approaches in ecotoxicological aquatic research. Environ. Pollut..

