# Supplementary material for: Lowest Environmentally Relevant Concentrations of Ionic Silver in Picograms per Liter Impair Life History Traits and Population Growth of Daphnia magna (Cladocera)
Source: J Xenobiot. 2026 Apr 2;16(2):60. doi: 10.3390/jox16020060 (PMC13117943; doi:10.3390/jox16020060)
Supplement: Supplementary file 1 [file jox-16-00060-s001.zip › jox-4198354-supplementary.pdf]

Journal of Xenobiotics

MDPI

Supplementary Materials: Lowest Environmentally Relevant Concentrations of Ionic Silver in Picograms per Liter Impair Life History Traits and Population Growth of *Daphnia magna* (Cladocera)

Jingyun Ding, Stefanie Krais, Zequn Li, Rita Triebskorn and Heinz-R. Köhler

Supplementary Table S1: Survival rates of *D. magna* in experimental series 1

Generations	Treatments	n	Survival Rate (%)
F0	C	20	100
	0.05	10	100
	0.5	9	90
	5	9	90
	15	8	80
F1	C-C	19	95
	0.05-0.05	9	90
	0.5-0.5	9	90
	5-5	9	90
	15-15	9	90
F2	C-C-C	19	95
	0.05-0.05-0.05	9	90
	0.5-0.5-0.5	9	90
	5-5-5	8	80
	15-15-15	7	70
F3	C-C-C-C	18	90
	0.05-0.05-0.05-0.05	9	90
	0.5-0.5-0.5-0.5	8	80
	5-5-5-5	8	80
	15-15-15-15	8	80
F1′	C-C-C-C-C	20	100
	0.05-0.05-0.05-0.05-C	9	90
	0.5-0.5-0.5-0.5-C	8	80
	5-5-5-5-C	9	90
	15-15-15-15-C	7	70
F2′	C-C-C-C-C-C	18	90
	0.05-0.05-0.05-0.05-C-C	10	100
	0.5-0.5-0.5-0.5-C-C	8	80
	5-5-5-5-C-C	9	90
	15-15-15-15-C-C	8	80
F3′	C-C-C-C-C-C-C	20	100
	0.05-0.05-0.05-0.05-C-C-C	9	90

	0.5-0.5-0.5-0.5-C-C-C	9	90
	5-5-5-5-C-C-C	9	90
	15-15-15-15-C-C-C	8	80

n: number of replicates

Supplementary Table S2: Survival rates of *D. magna* in experimental series 2

Generations	Treatments	n	Survival rate (%)
F0	C	20	100
	15	8	80
F1	C-C	19	95
	15-C	7	70
	15-15	9	90
F2	C-C-C	19	95
	15-C-C	8	80
	15-C-15	9	90
	15-15-C	8	80
	15-15-15	7	70
F3	C-C-C-C	18	90
	15-C-C-C	9	90
	15-C-C-15	9	90
	15-C-15-C	8	80
	15-C-15-15	6	60
	15-15-C-C	9	90
	15-15-C-15	9	90
	15-15-15-C	8	80
	15-15-15-15	8	80

n: number of replicates
